# Improving the Hip Fracture Risk Prediction with a Statistical Shape-and-Intensity Model of the Proximal Femur

**DOI:** 10.1007/s10439-022-02918-z

**Published:** 2022-01-19

**Authors:** Alessandra Aldieri, Pinaki Bhattacharya, Margaret Paggiosi, Richard Eastell, Alberto Luigi Audenino, Cristina Bignardi, Umberto Morbiducci, Mara Terzini

**Affiliations:** 1 PolitoBIOMed Lab, Department of Mechanical and Aerospace Engineering, Politecnico di Torino, Corso Duca degli Abruzzi, 24, 10129 Turin, Italy; 2 Department of Mechanical and Aerospace Engineering, Politecnico di Torino, Turin, Italy; 3 INSIGNEO Institute for in silico Medicine, University of Sheffield, Sheffield, UK; 4 Department of Mechanical Engineering, University of Sheffield, Sheffield, UK; 5 Academic Unit of Bone Metabolism, University of Sheffield, Sheffield, UK

**Keywords:** Femur fracture, Osteoporosis, Fracture risk assessment, PLS, SSIM

## Abstract

Severe predictions have been made regarding osteoporotic fracture incidence for the next years, with major economic and social impacts in a worldwide greying society. However, the performance of the currently adopted gold standard for fracture risk prediction, the areal Bone Mineral Density (aBMD), remains moderate. To overcome current limitations, the construction of statistical models of the proximal femur, based on three-dimensional shape and intensity (a hallmark of bone density), is here proposed for predicting hip fracture in a Caucasian postmenopausal cohort. Partial Least Square (PLS)-based statistical models of the shape, intensity and their combination were developed, and the corresponding modes and components were identified. Logistic regression models using the first two shape, intensity and shape-intensity PLS components were implemented and tested within a 10-fold cross-validation procedure as predictors of hip fracture. It emerged that (1) intensity components were superior to shape components in stratifying patients according to their fracture status, and that (2) a combination of intensity and shape improved patients risk stratification. The area under the ROC curve was 0.64, 0.85 and 0.92 for the models based on shape, intensity and shape-intensity combination respectively, against a 0.72 value for the aBMD standard approach. Based on these findings, the presented methodology turns out to be promising in tackling the need for an enhanced fracture risk assessment.

**Supplementary Information:**

The online version contains supplementary material available at 10.1007/s10439-022-02918-z.

## Introduction

Osteoporosis is a metabolic disease very common in older adults, entailing bone mass reduction and micro-architectural deterioration eventually leading to bone fracture.^38^ Globally, 9 million fragility fractures were estimated to occur in 2000, including 1.6 million hip fractures,^22^ with predictions suggesting this number will increase 3 to 4-fold by 2050.^12,18^ Osteoporotic fractures are a burden for the public health, costing 37 billion euros in EU and 16 billion dollars in USA.^7,19^ Among others, hip fractures represent one of the most common and serious osteoporosis-related fractures, causing severe morbidity and mortality.^20,33^ Hip fracture incidence increases exponentially with age, mainly due to progressive loss of bone mass, and structural and material deterioration of trabecular and cortical bone, combined with other non-skeletal factors.^8^ Considering the adverse impact of hip fracture on patients’ life, the identification of individuals at high risk is pivotal, since it is the gateway to fracture prevention. According to the World Health Organisation (WHO) it is the areal Bone Mineral Density (aBMD), measured through Dual X-rays Absorptiometry (DXA) at the proximal femur or lumbar spine, that currently supports the diagnosis of osteoporosis and the indication for treatment.^11,23–25^ However, aBMD is a surrogate marker of bone strength able to, at best, partially capture the influence that factors such as bone shape, amount of cortical and trabecular bone and the overall bone density spatial distribution have on fracture risk.^21,29^ As a consequence, the aBMD performance in identifying fracture from non-fracture cases is moderate, characterized by sensitivity and specificity settling at about 55% and 75% respectively.^11,23^

In light of the moderate performance of aBMD as fracture predictor and aiming at overcoming the current critical issues, research into more advanced and diversified diagnostic methods has been fostered. In particular, a number of measurable anatomical features extracted from DXA through the Hip Structural Analysis (HSA) have been tested as additional hip fracture predictors,^16^ but no substantial improvement in the aBMD predictive power was obtained.^4^ In parallel, the adoption of patient-specific Finite Element (FE) models as part of more comprehensive multiscale models was explored,^2,6,44^ with the final aim of orchestrating together the main factors involved in the risk of hip fracture. Although they were shown to enhance the fracture risk prediction, the cost-effectiveness of such multiscale modelling strategies, and their translation to clinics as well, has not been definitively proven yet.^43^

Very recently, the combined effect of proximal femur shape and BMD distribution on hip fracture risk has been promisingly investigated through statistical models.^4,9,15,45^ Statistical models, by fully exploiting the information contained in clinical images,^41^ have led to a substantial enhancement of fracture risk prediction.^35^ Commonly, the construction of most of such statistical models is based on dimensionality reduction as obtained through Principal Component Analysis (PCA).^4,9,15,41^ However, PCA-based strategies can only identify the most variable anatomical or intensity features, without any connection to a known fracture risk, thus limiting the identification of actual fracture predictors. To overcome this limitation, very recently a statistical model based on both anatomical and densiometric information contained in DXA images and relying on a Partial Least Square (PLS) algorithm^34^ has been successfully proposed for hip fracture prediction.^1^ In detail, the PLS-based shape-and-intensity statistical model was effective in identifying the main anatomical and intensity features simultaneously related to femoral strength and ultimately to fracture risk. The PLS algorithm relies on covariance maximisation and allows the identification of the directions where the covariance between two different quantities (e.g., shape/intensity and fracture risk) is maximal. In fact, the adoption of this approach turned out to be more suitable than PCA when dealing with fracture ‘status’ classification.^5^

In this study a PLS-based shape-and-intensity statistical model based on Computed Tomography (CT) images is retrospectively applied to a post-menopausal cohort, aiming at: (1) exploring the relationship between shape and intensity and their respective role in affecting hip fracture risk; (2) evaluating the improvement in hip fracture discrimination achieved using logistic regression classifiers built on the main PLS algorithm outcomes (the PLS components).

## Materials and Methods

### Subjects

The statistical models were developed based on a cohort of 100 Caucasian women who were at least 5 years post menopause, 50 of whom (55–89 years old) had suffered from a hip fracture and 50 selected to be pair matched in terms of age, height, and weight. Details of the cohort are extensively reported elsewhere^46^ and presented in Table S4 (Supplementary Material). Due to incomplete CT data, 7 subjects were excluded from the analysis (Table 1). Informed written consent was obtained for all participants. The patients underwent QCT scans (LightSpeed 64 VCT, GE Medical Systems at 120 KVp/150 mA). For each acquired subject the scanned region included from above the femoral head to 3.5 cm below the lesser trochanter. For subjects having experienced a fracture, the contralateral femur was used for analysis, assuming that similar pathological or fracture-prone shape and density features were exhibited by the two femurs (none of the patients suffered from pathologies such as bone tumours or dysplasia). A data summary of the subjects here considered is reported in Table 1.

**Table 1:** Subjects’ clinical data.

	Fractured patients (46 subjects)		Non-fractured patients (47 subjects)	
	Mean	SD	Mean	SD
Age	75.4	9.1	74.6	9.0
Mass (kg)	63.2	15.2	64.2	12.2
Height (cm)	158.8	6.7	157.8	5.7
aBMD (g/cm^2^)	0.696	0.148	0.820	0.146

### Femur anatomical shape representation

The proximal femur geometries, reconstructed from CT images as extensively reported elsewhere,^32^ were realigned based on a two-steps procedure: (1) alignment to a reference shape (i.e., the smallest shape identified in the cohort) based on the femoral head centre and neck-shaft axis; (2) further alignment procedure based the Iterative Closest Point (ICP) algorithm.^10^

**Figure 1:**
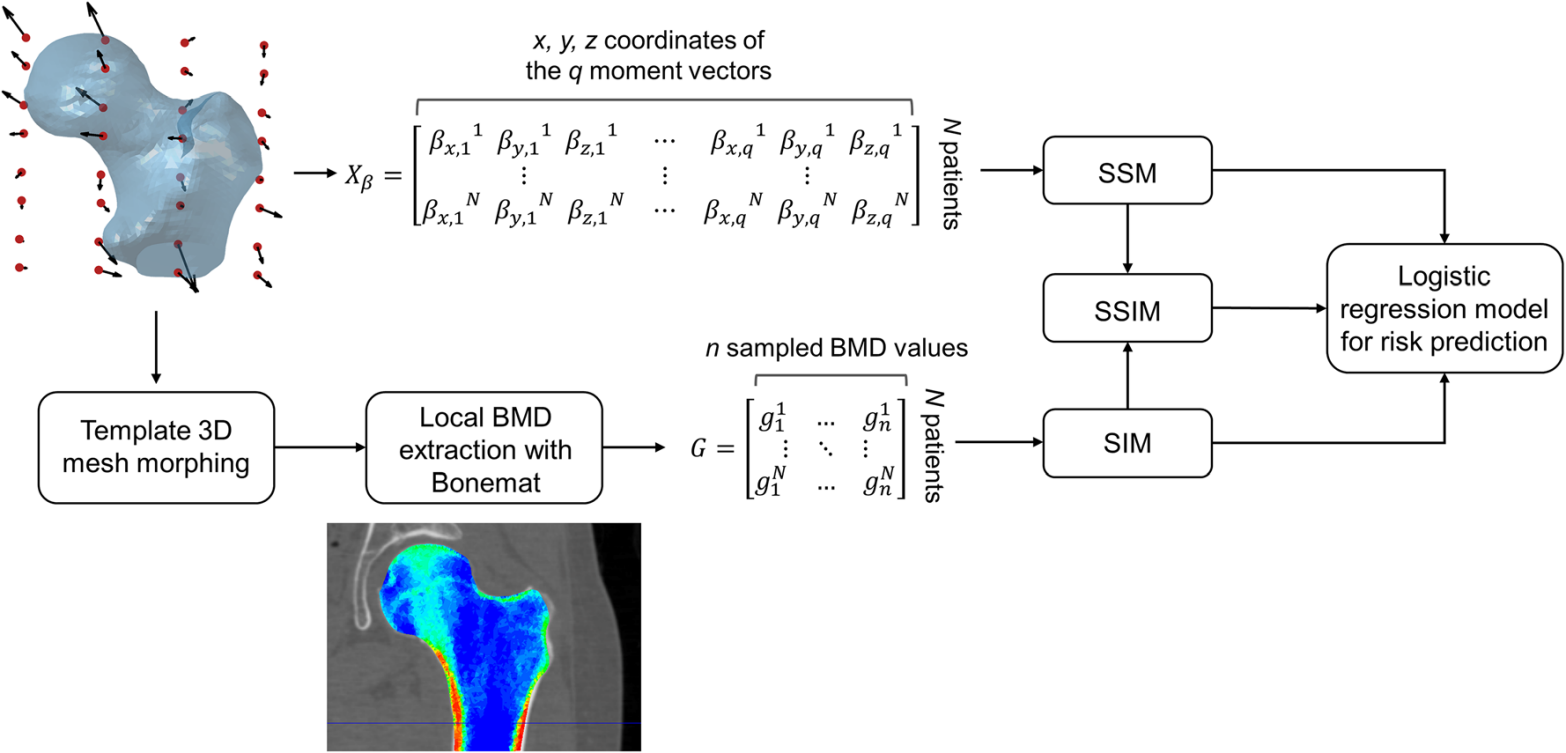
A schematic representation of the whole workflow: the moment vectors basis of the non-parametric shape representation were gathered into the ${X}_{\beta }$ matrix, so that the SSM of the proximal femur was built. The moment vectors were also employed to morph a 3D mesh from the template onto each subject, and Bonemat was used to extract element-specific BMD values. These latter, gathered in the matrix *G*, allowed the SIM construction. Eventually, a SSIM could be built combining the SSM and SIM PLS components. Logistic regression models were then implemented based on the three different statistical models for hip fracture risk prediction.

At the end of the alignment procedure the proximal femur models were processed according to a recently proposed strategy^1^ based on a non-parametric shape representation, which prevents the need for landmarking and has been implemented in the open source code Deformetrica^14^ (https://www.deformetrica.org/)**.** As extensively described elsewhere^1^ and briefly summarized in the Supplementary Material, this approach allowed to obtain the template, i.e., the mean anatomical shape of the population, together with the so called moment vectors for each patient, quantities which gather the patient-specific shape features. Technically, each *i*th femur shape in the cohort was described by a set of vectors, called moment vectors ${\beta }^{i}$, centred at a common grid of control points and defining the deformations the template should experience to match each subject-specific femur shape (Figs. 1 and S1 in the Supplementary Material). Based on the here adopted non-parametric shape representation, the subject-specific set of moment vectors can then be regarded as surrogates of the subject-specific femur shape features. The generic *i*th (*i* = 1…*N*, where *N* is the number of subjects) patient-specific moment vectors were expressed as follows:

1 $${\beta }^{i}={\beta }_{x 1}^{i},{\beta }_{y 1}^{i},{\beta }_{z 1}^{i},\ldots ,{\beta }_{x q}^{i},{\beta }_{y q}^{i},{\beta }_{z q}^{i},$$

where *i* refers to the subject and *q* to the total number of moment vectors. Using the patient-specific moment vector representation of Eq. (1), the (*N* × 3*q*) shape matrix ${X}_{\beta }$ (Fig. 1) was built by storing each ${\beta }^{i}$ in the *i*th row of the matrix.

### Statistical Models

Taking advantage of the anatomical and densitometric information contained in CT images, a Statistical Shape Model (SSM), a Statistical Intensity Model (SIM) and a Statistical Shape-Intensity Model (SSIM), combining shape and intensity together, were built. A schematic of the workflow is presented in Fig. 1. Aiming to extract fracture-prone shape and intensity features to achieve subjects classification, a fracture status binary array of the analysed cohort was generated where, for each patient in the cohort, 0 indicated non-fracture, 1 indicated fracture. The statistical models were all built performing a PLS-based dimensionality reduction,^1,34^ which allowed to identify the directions (also called modes) of maximal covariance between the input matrix of either shape, intensity or their combination and the fracture status array.^5,30^ PLS algorithm was adopted here because of its recently emerged effectiveness in identifying fracture-related shape and intensity features.^1^ Technical details on PLS algorithm are summarized in Supplementary Material.

The SSM, SIM and SSIM are to be interpreted as representations of either the shape, the intensity or the combination of the two, here generically indicated by ${x}^{i}$ for the *i*th patient, according to:

2 $${x}^{i}=\overline{x }+\sum_{j=1}^{m}{t}_{j}^{i}{p}_{j},$$

where $\overline{x }$ represents the average value of the generic variable *x*, the *p*_*j*_ vectors (i.e., the PLS modes) define the new space of representation of the original variables *x* where their covariance with the fracture status is maximised, and the coefficients ${t}_{j}^{i}$ (i.e., the PLS components, obtained projecting the original variables onto the PLS modes) measure the relevance of each *j*th mode in the representation of the *i*th patient. The PLS components ${t}_{j}^{i}$ thus offer the representation of the shape (SSM), of the BMD distribution (SIM) or of the combination of the two (SSIM) in a new space of maximal covariance with the fracture status and were therefore employed for the subsequent analysis.

The SSM was built by applying PLS to the *N* × 3*q* shape matrix ${X}_{\beta }$ and to the fracture status array (Fig. 1). The SIM, based upon Bone Mineral Density (BMD) distribution features, was built up as follows. The template (i.e., the average shape) was first meshed with tetrahedral elements (1.5 mm edge length) using Hypermesh (Altair Engineering Inc., Troy, USA). Subsequently, the previously obtained subject-specific moment vectors were used to morph the template three-dimensional mesh onto each subject’s femur shape, so that iso-topological meshes would allow a consistent sampling of the local BMD across the subjects, as explained in the following. After calibrating the grey levels in the images to the corresponding bone density values using the five-sample calibration phantom visible in the images of every participant (hydroxyapatite density range: 0–200 mg/cm^3^; Mindways Software, Inc., Austin, TX), the freeware software Bonemat (http://www.bonemat.org/) was used to map on the individual tetrahedral meshes the density values derived from individual CT images.^40^ In analogy to femur shapes, a *N* × *n* (with *n* the number of elements and therefore of density local values) intensity matrix *G* was built gathering the patient-specific intensity vectors (i.e., the whole sets of the elements-based density values, Fig. 1). Finally, the SIM was obtained by applying a PLS-based strategy to the intensity matrix *G* and the fracture status array.

In order to minimize the impact of possible outliers on the PLS-based strategy, both for the SSM and SIM construction the presence of outliers was assessed using the Cook’s distance within a leave-one-out approach, as suggested elsewhere.^10^

With the purpose of unifying the independent SSM and SIM approaches, a SSIM, intended to account for the shape and density distributions together, was also built.^1,13,39^ Specifically, the SSIM construction was based on the previously determined shape and intensity PLS components (Eq. (2)): the shape and intensity components matrices were concatenated in a unique matrix. Hence, PLS was applied to the latter and to the fracture status array (details are presented in the Supplementary Material).

### Prediction of Fracture Risk

The statistical models (the SSM, SIM and SSIM) were used to predict the subject-specific hip fracture risk. A regression analysis based on the use of a logistic function was carried out between the PLS components, taken as independent predictors, and the fracture status, taken as binary dependent variable. More in detail, the first two PLS components of either SSM, SIM or SSIM were considered here as independent predictors, leading to three distinct predictive models. The prediction power of these logistic regression models based on the SSM, SIM and SSIM output variables was compared with that of the separate regression model using aBMD as the independent variable and the fracture status as binary dependent variable, aBMD being currently recognized as the gold standard for patients’ risk stratification.

Initially, a logistic regression model was built considering the whole available cohort, therefore relying on the statistical models built considering all the 93 patients. However, since a test set would be necessary for the predictive performance of the approach to be assessed, a *k*-fold cross-validation procedure was adopted according to the following standard steps: (1) the whole cohort was divided into *k* subsets; (2) different statistical models were built *k* times, each *k*th time leaving the *k*th subset out from the training set; (3) *k* different logistic regression models were trained, each using the *k*th statistical model outcomes; (4) each *k*th logistic regression model was tested taking advantage of the *k*th cohort subset left out from the training set. Specifically, herein a 10-fold cross-validation was applied by randomly dividing the whole cohort in 10 groups, estimating fracture risk for a test set while maximising the number of subjects in the training set as well. PLS was performed and the logistic regression models were trained and tested 10 different times, predicting the fracture risk for the subjects included in the test group. Each group consisted, as far as possible, of the same number of fractured and non-fractured patients, to assure training sets properly balanced. Then, aiming to compare the diagnostic value of the different logistic regression models, the respective Receiver Operating Characteristic (ROC) curves were plotted and the Area Under Curve (AUC) was computed for each ROC curve.

## Results

No outliers could be identified in any of the constructed statistical models.

**Figure 2:**
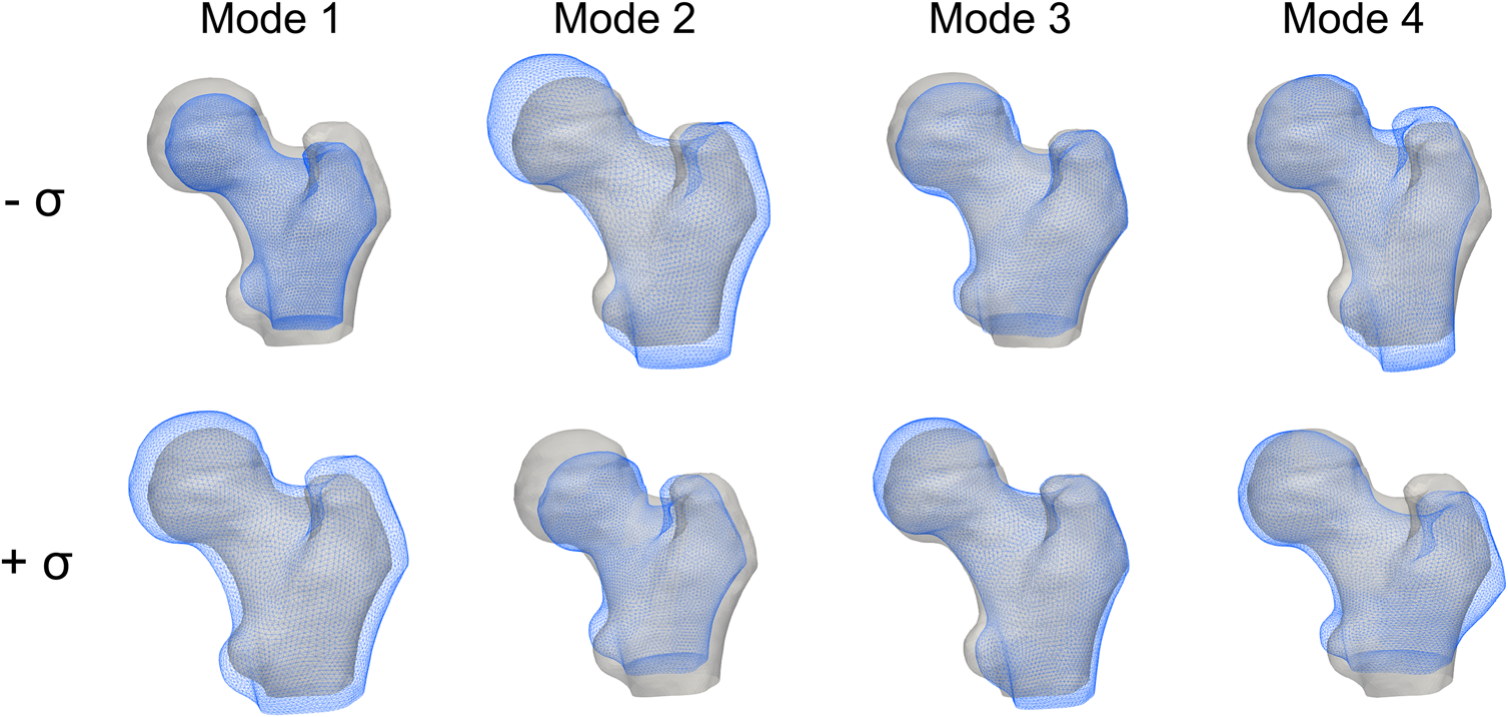
The first four PLS shape modes, shown as deformation of the template along each mode between $-\sigma , +\sigma$, where ${\sigma }^{2}$ represents the mode variance. The template is displayed in grey.

**Figure 3:**
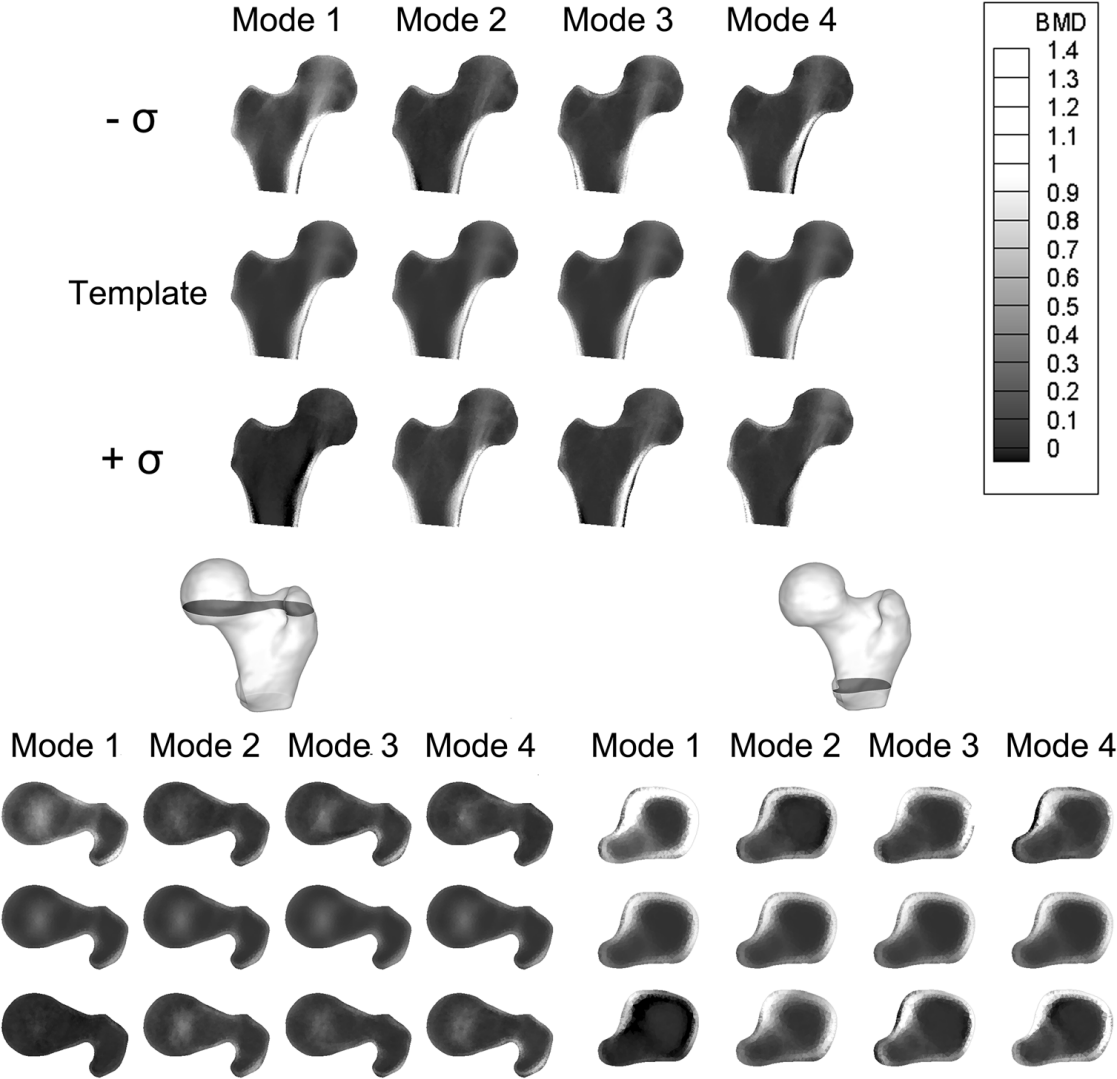
The first four PLS intensity modes, shown as intensity variations from the template along each mode between $-\sigma , +\sigma$, where ${\sigma }^{2}$ represents the mode variance. Above, a proximal femur cross-section in the frontal plane is presented, while below two other different cross-sections, taken in the transversal plane, are displayed. In each of the three case, the second row always shows the template, the first $-\sigma$ variation, the third the $+\sigma$ variation.

**Figure 4:**
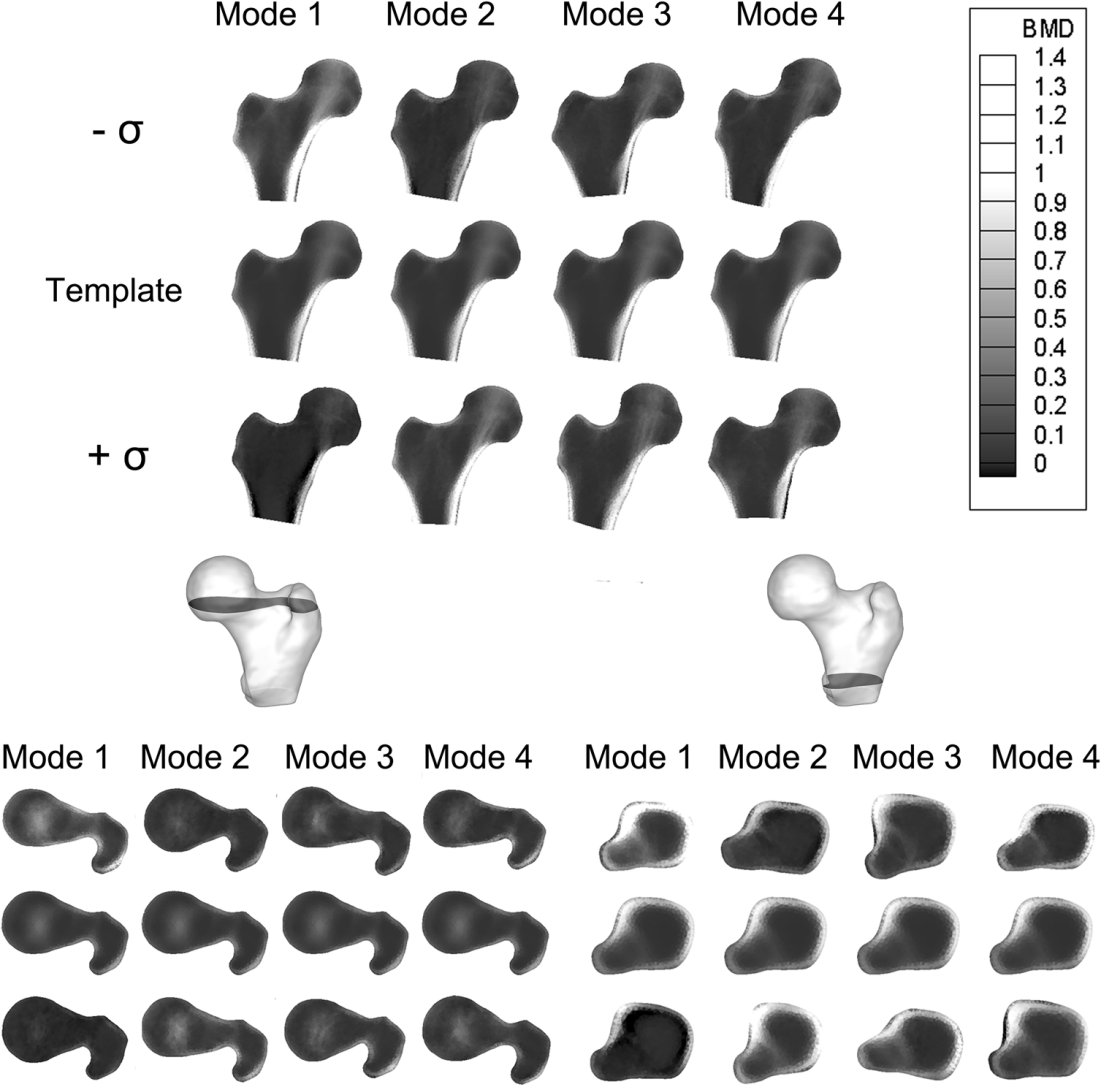
The first four PLS shape-intensity modes, shown as shape and intensity variations from the template along each mode between $-\sigma , +\sigma$, where ${\sigma }^{2}$ represents the mode variance. Above, a proximal femur cross-section in the frontal plane is presented, while below two other different cross-sections, taken in the transversal plane, are displayed. In each of the three case, the second row always shows the template, the first the $-\sigma$ variation, the third the $+\sigma$ variation.

Among the three models here introduced, the SSM was the most compact one, with 22 modes able to explain more than 90% of the total variation in shape. The first four shape modes, able to explain 43% of the total shape variation, are displayed as deformations of the template in Fig. 2. It can be noticed by visual inspection that size, inclination and length of the neck as well as the greater trochanter represent the main shape features involved (Fig. 2), with Mode 1 emphasising the already emerged^31^ association between a larger bone and an higher susceptibility to fracture. The SIM model was less compact than the SSM: 67 modes were needed to account for at least 90% variation in the BMD distribution, the first four modes accounting for the 37% of it (Fig. 3). The first intensity mode, alone explaining 33% of the total variation in the BMD, clearly captures the overall variation in bone mass as well as in cortex thickness, the latter visible also in the subsequent modes. The whole femur bone mass variation also represents the main feature of the first SSIM mode (Fig. 4) which, simultaneously accounting for both intensity and shape, also captures variations in the cross-sections, consistent with the protective role that an increased cross-sectional moment of inertia is known to play on cortical stability.^26^

The other SSIM modes display variations in cortical thickness and cortical bone distribution, as well as in neck inclination and neck cross-sectional area (modes 3 and 2, respectively) as far as changes in the shape are concerned. While 65 modes sufficed to explain at least 90% of the total variation in shape-and-intensity, the first four modes accounted for 33% of the total combined variation. Fig. S2 in the Supplementary Material provides a comparative overview of the percentage variation explained by the modes in the three different statistical models.

**Figure 5:**
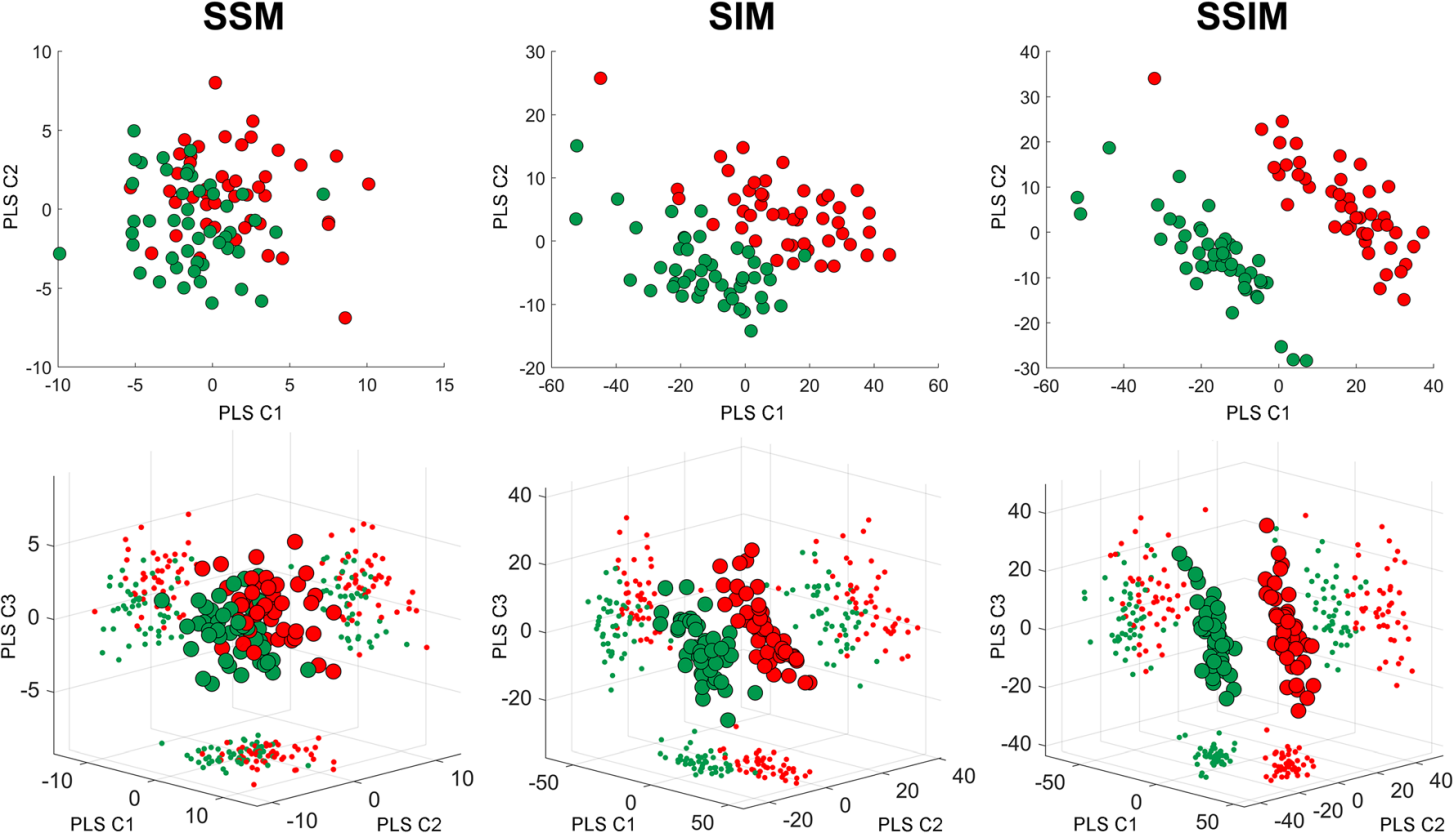
Scatter plots of the PLS components corresponding to the first two (upper panel) and three (lower panel) modes for the SSM, SIM and SSIM. The PLS components of the fractured and non-fractured patients are depicted in red and green respectively. The plot refers to the statistical models built on the full cohort.

The capability of PLS components to identify hip fracture status is highlighted in Fig. 5, where the scatter plots of the PLS components in the space defined by the first two (Fig. 5, upper panel) and three (Fig. 5, lower panel) modes for shape, intensity and shape-and-intensity are presented. It emerges by visual inspection of Fig. 5 that: (1) the capability of PLS components of discriminating between fracture and non-fracture status groups gets more substantial if the intensity (SIM) and shape-and-intensity (SSIM) modes are considered; (2) PLS components in the space defined only by the first and the second shape-and-intensity (SSIM) PLS modes adequately discriminate between fractured and non-fractured subjects.

In Fig. S3 in the Supplementary Material the patient-specific aBMD values for the fracture and control cases are presented analogously.

**Figure 6:**
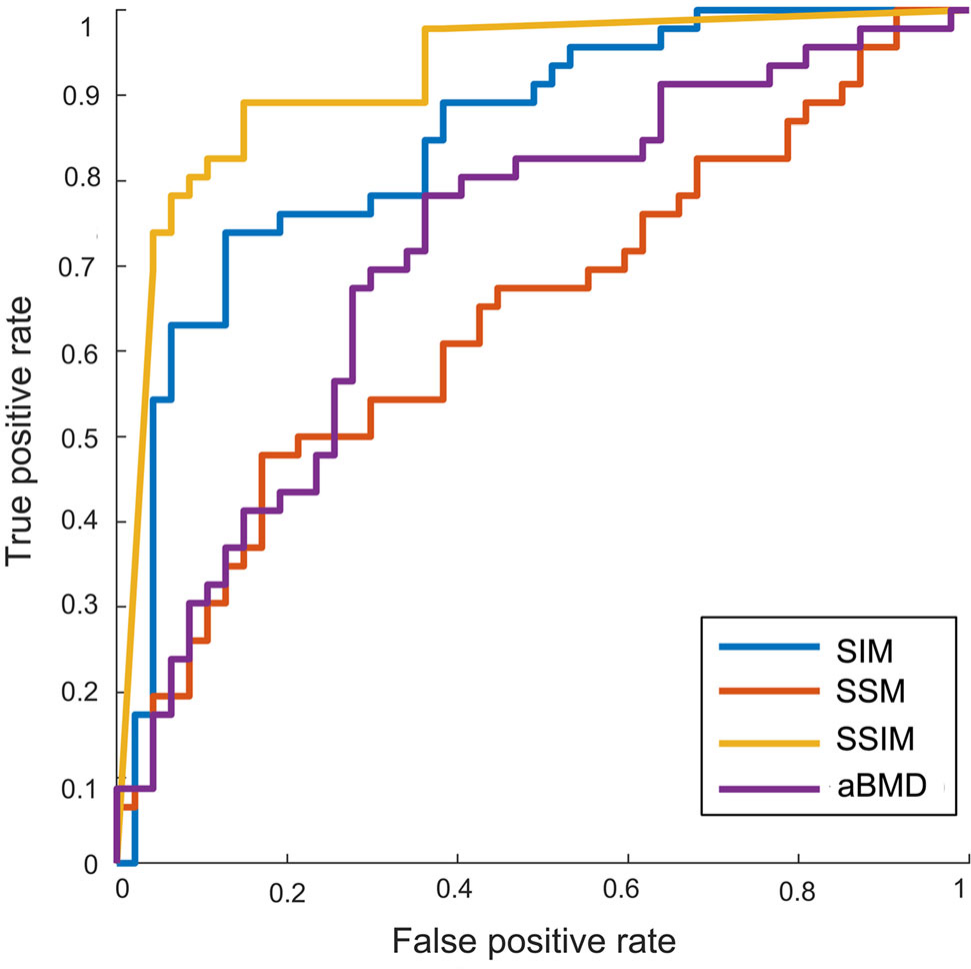
ROC curves as calculated following the 10-fold cross-validation for the different fracture risk considered: the SSM-, SIM-, SSIM-based PLS components related to the first two modes and the aBMD as extracted at the femoral neck.

**Figure 7:**
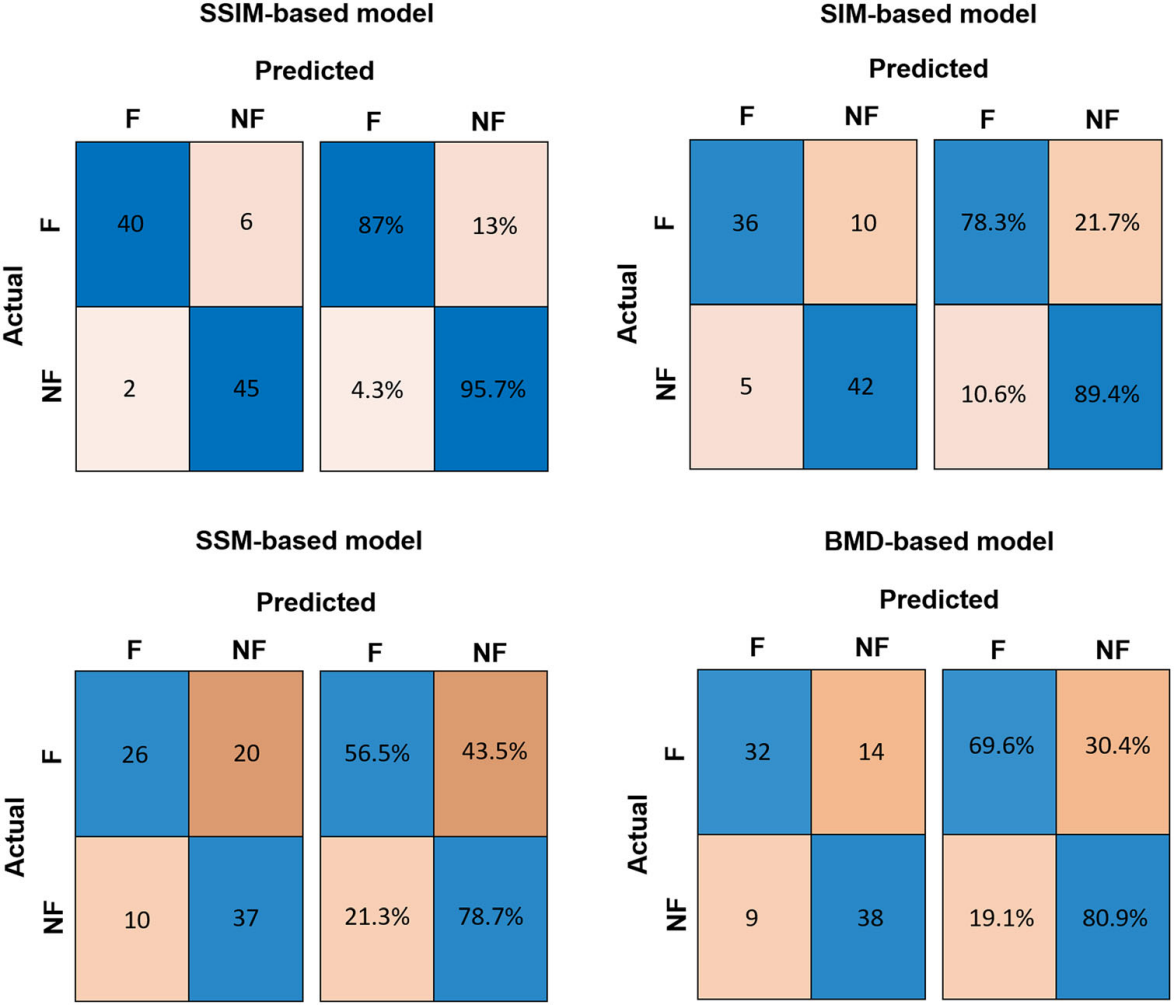
Confusion matrices related to the classification of the patients as fractured or non-fractured for the SSM, SIM, SSIM and BMD-based regression models.

The analysis of the ROC curves (Fig. 6), performed on the 10 cross-validation folds, highlights associated AUC values equal to 0.64 (95% CI 0.56–0.73) for the SSM, 0.85 (95% CI 0.78–0.91) for the SIM, 0.92 (95% CI 0.88–0.96) for the SSIM, and 0.72 (95% CI 0.61–0.83) for the aBMD, the gold standard for hip fracture risk assessment. Notably, the ROC curves of SIM and SSIM present AUC values sensibly higher than those of aBMD, suggesting an improved diagnostic ability of these two statistical models with respect to the gold standard. The confusion matrices related to the 10-fold cross-validation procedure for patient classification are provided in Fig. 7. The logistic regression models coefficients are provided in the Supplementary Material (Tables S1–S3).

## Discussion

In the last decades, efforts have been put in the improvement of osteoporotic hip fractures prediction.^6,8,15,16,29,32,45^ In light of the moderate predictive potency of the aBMD, the predictive power of the proximal femur anatomical features has been tested, although it turned out to only slightly enhance aBMD-derived predictions.^17^ More recently patient-specific CT-based computational models have been demonstrated to improve the predictive performance of aBMD,^2,6^ although they have not become part of the clinical practice yet.^43^ While aBMD represents a unique average density value, the question remains whether looking at the BMD locally could support fracture risk prediction.

Within this multifaceted context, this study tested CT-based statistical models built on the shape and BMD distribution of a post-menopausal cohort as predictors of hip fracture risk. Based on a recent study^1^ highlighting the huge potential of PLS-based statistical models algorithms in identifying hip fracture-prone features, here PLS-based shape, intensity and shape-and-intensity statistical models were retrospectively applied to a large cohort of subjects with a known fracture status with the main goal of improving the proximal femur fracture risk assessment. PLS was adopted instead of PCA due to its ability to consider two different variables simultaneously for the covariance maximisation, which herein allowed the direct identification of fracture-prone features. Being a supervised technique, PLS can accomplish considerable improvement when multivariate predictive models are the key area of application.^27,36^

Among the findings of this study, shape features alone were demonstrated not to satisfactorily predict the risk of fracture (Fig. 5) also in the case of a PLS-based strategy. In accordance to previous studies,^1,41^ that was not surprising, given the relatively simple and standard proximal femur shape on one hand, and the moderate role of geometric attributes highlighted by similar works^1,16,17^ on the other. Notably, the performance of the PLS-based statistical model based only on shape was sensibly enhanced when a shape-intensity combined approach was applied (Fig. 5). A similar outcome was somehow anticipated by a recent study where, in spite of the limited role of the shape, the emerged association of the combination of shape and intensity with an estimated femoral strength was stronger than intensity alone.^1^ Summarizing, the ROC curves analysis highlighted that: (1) the discrimination power of the SSM-based model was even lower than the aBMD-based model, even though the two turned out to be not significantly different; (2) the discrimination power of SIM and SSIM was sensibly higher than the aBMD-based model, with the SSIM-related AUC settling at a value of 0.92 against the 0.72 AUC value obtained from the aBMD-based model. It must be noticed that the three different predictive models were built based on the same number of modes. Although two shape-intensity modes were actually sufficient to explain 90% of the variation in the fracture status, that was not true for the intensity and shape modes. In fact, four intensity and sixteen shape modes would be necessary to account for the same amount of variation in the fracture status. If new logistic regression models are built based on the SIM and SSM by increasing the number of predictor variables to four and sixteen respectively, the AUC related to the 10 cross-validation folds reach 0.90 and 0.84 for the SIM and SSM respectively, which still do not outperform the SSIM-related outcome.

The here obtained PLS-based only shape and only intensity modes can be compared to the corresponding PCA-based modes presented elsewhere^41^ and based on the same cohort, revealing similar shape and intensity features. Nonetheless, it can be observed that the here developed PLS-based predictive models demonstrate a markedly improved level of performance with respect to the PCA-based approach,^41^ substantiating PLS superiority to the traditionally employed PCA when discrimination is of concern. From this perspective, the statistical modelling-based approach proposed in this study was proven to be effective in substantially enhancing fracture risk prediction. Of course, further studies based on comprehensive training and test sets will be necessary to support the implementation of statistical models in the clinical practice. If the results obtained using the SSIM-based components are promising, the presented study faces possible limitations. First, the afore-mentioned retrospective nature of the analysed cohort. In this framework, the use of a retro-prospective cohort (i.e., a cohort who is followed up from the time of the CT on, so that information about the fracture status is available) would be extremely valuable. Nonetheless, the prevalence of hip fractures in the general population is low, and the absolute risk of fracture is typically evaluated over 10 years. Therefore, a true prediction accuracy is hard to be evaluated through an observational study and a retrospective stratification accuracy, as done here, is often adopted as the most feasible approach.

In addition, factors related to the patient history other than shape and mechanical properties of the proximal femur (e.g., drugs, fracture history, tobacco alcohol consumption,^42^ fall mechanics^28^ and falling probability^37^ among others) were here not considered. Eventually, it should be recalled that CT does not currently represent the standard imaging technique adopted for osteoporosis diagnosis purposes: this might seem to hinder the clinical feasibility of the presented approach. Nevertheless, the performance assured by the presented statistical models might contribute to foster CT adoption for fracture risk prediction in the future. In analogy to the other two well-known CT-based technologies, PCA and FE, the presented PLS-based approach would require CT images segmentation. In this regard, PLS and PCA are equally demanding in terms of costs and computational effort: once the predictive model is identified based on a consistent cohort which is required a priori, any new patient could be registered onto the template and classified according to his components. FE, instead, would not necessitate a cohort a priori, but would require to run patient-specific simulations, with computational costs currently not addressable in a clinical framework and uncertainties related to the fall-induced loading and boundary conditions. In this connection, a FE-based study on the same cohort object of this study achieved a maximum AUC of 0.82, lower than the here presented SSIM-based AUC.^3^ The uncertainties related to the imposed boundary conditions in the FE simulations, not included in the SSIM-based predictive model, might be responsible for this differences in the obtained AUC.

In conclusion, the findings of this study demonstrate the improved predictive capability of a PLS-based statistical modelling approach combining shape and density information, and simultaneously brings evidence of PLS superiority to PCA when classification is required. The findings of this study also corroborate the hypothesis that density and its distribution throughout the bone play a more relevant role than bone shape in fracture determination, confirming the results of previous studies.^41^ In a translational perspective, the proposed approach advocates the promising possibility to be adopted in the context of fracture risk prediction. In this light, an adequately large and exhaustive training set would make the development of a consistent SSIM possible. Once extracted from clinical images, each new patient’s shape and intensity features could then be projected on the space defined by the shape-intensity PLS modes. The corresponding PLS components could eventually be used as predictors within the previously trained logistic regression model.

## Supplementary Information

Below is the link to the electronic supplementary material.

Supplementary file1 (PDF 857 kb)

## References

[1] Aldieri, A., M. Terzini, A. L. Audenino, C. Bignardi, and U. Morbiducci. Combining shape and intensity DXA-based statistical approaches for osteoporotic HIP fracture risk assessment. *Comput. Biol. Med.* 127:104093, 2020. 10.1016/j.compbiomed.2020.104093 33130436

[2] Altai Z, Montefiori E, van Veen B, Paggiosi MA, McCloskey EV, Viceconti M, Mazzà C, Li X (2021). Femoral neck strain prediction during level walking using a combined musculoskeletal and finite element model approach. PLoS ONE.

[3] Altai Z, Qasim M, Li X, Viceconti M (2019). The effect of boundary and loading conditions on patient classification using finite element predicted risk of fracture. Clin. Biomech..

[4] Baker-LePain JC, Luker KR, Lynch JA, Parimi N, Nevitt MC, Lane NE (2011). Active shape modeling of the hip in the prediction of incident hip fracture. J. Bone Miner. Res..

[5] Barker M, Rayens W (2003). Partial least squares for discrimination. J. Chemom..

[6] Bhattacharya P, Altai Z, Qasim M, Viceconti M (2019). A multiscale model to predict current absolute risk of femoral fracture in a postmenopausal population. Biomech. Model. Mechanobiol..

[7] Blume SW, Curtis JR (2011). Medical costs of osteoporosis in the elderly Medicare population. Osteoporos. Int..

[8] Bouxsein ML, Zysset P, Glüer CC, McClung M, Biver E, Pierroz DD, Ferrari SL (2020). Perspectives on the non-invasive evaluation of femoral strength in the assessment of hip fracture risk. Osteoporos. Int..

[9] Bredbenner TL, Mason RL, Havill LM, Orwoll ES, Nicolella DP (2014). Fracture risk predictions based on statistical shape and density modeling of the proximal femur. J. Bone Miner. Res..

[10] Bruse JL, McLeod K, Biglino G, Ntsinjana HN, Capelli C, Hsia TY, Sermesant M, Pennec X, Taylor AM, Schievano S, Taylor A, Giardini A, Khambadkone S, de Leval M, Bove E, Dorfman A, Baker GH, Hlavacek A, Migliavacca F, Pennati G, Dubini G, Marsden A, Vignon-Clementel I, Figliola R, McGregor J (2016). A statistical shape modelling framework to extract 3D shape biomarkers from medical imaging data: assessing arch morphology of repaired coarctation of the aorta. BMC Med. Imaging.

[11] Chen SJ, Chen YJ, Cheng CH, Hwang HF, Chen CY, Lin MR (2016). Comparisons of different screening tools for identifying fracture/osteoporosis risk among community-dwelling older people. Medicine.

[12] Cooper C, Campion G, Melton LJ (1992). Hip fractures in the elderly: a world-wide projection. Osteoporos. Int..

[13] Cootes T. F., G. J. Edwards, and C. J. Taylor. Active appearance models. In: Computer Vision—ECCV’98. ECCV 1998, edited by H. Burkhardt and B. Neumann. Lecture Notes in Computer Science, vol. 1407. Berlin: Springer, 1998. 10.1007/BFb0054760.

[14] Durrleman S, Prastawa M, Charon N, Korenberg JR, Joshi S, Gerig G, Trouvé A (2014). Morphometry of anatomical shape complexes with dense deformations and sparse parameters. Neuroimage.

[15] Goodyear SR, Barr RJ, McCloskey E, Alesci S, Aspden RM, Reid DM, Gregory JS (2013). Can we improve the prediction of hip fracture by assessing bone structure using shape and appearance modelling?. Bone.

[16] Gregory JS, Aspden RM (2008). Femoral geometry as a risk factor for osteoporotic hip fracture in men and women. Med. Eng. Phys..

[17] Gregory JS, Testi D, Stewart A, Undrill PE, Reid DM, Aspden RM (2004). A method for assessment of the shape of the proximal femur and its relationship to osteoporotic hip fracture. Osteoporos. Int..

[18] Gullberg B, Johnell O, Kanis JA (1997). World-wide projections for hip fracture. Osteoporos. Int..

[19] Hernlund E, Svedbom A, Ivergård M, Compston J, Cooper C, Stenmark J, McCloskey EV, Jönsson B, Kanis JA (2013). Osteoporosis in the European Union: Medical management, epidemiology and economic burden: a report prepared in collaboration with the International Osteoporosis Foundation (IOF) and the European Federation of Pharmaceutical Industry Associations (EFPIA). Arch. Osteoporos..

[20] Ip TP, Leung J, Kung AWC (2010). Management of osteoporosis in patients hospitalized for hip fractures. Osteoporos. Int..

[21] Jazinizadeh F, Quenneville CE (2021). 3D analysis of the proximal femur compared to 2D analysis for hip fracture risk prediction in a clinical population. Ann. Biomed. Eng..

[22] Johnell O, Kanis JA (2006). An estimate of the worldwide prevalence and disability associated with osteoporotic fractures. Osteoporos. Int..

[23] Kanis J (2002). Diagnosis of osteoporosis and assessment of fracture risk. Lancet.

[24] Kanis, J. A. on behalf of the World Health Organization Scientific Group. Assessment of osteoporosis at the primary health-care level. Technical Report. World Health Organization Collaborating Centre for Metabolic Bone Diseases, University of Sheffield, 2007.

[25] Kanis JA, Cooper C, Rizzoli R, Reginster J-Y (2019). European guidance for the diagnosis and management of osteoporosis in postmenopausal women. Osteoporos. Int..

[26] Kaptoge S, Beck TJ, Reeve J, Stone KL, Hillier TA, Cauley JA, Cummings SR (2008). Prediction of incident hip fracture risk by femur geometry variables measured by hip structural analysis in the study of osteoporotic fractures. J. Bone Miner. Res..

[27] Maitra S, Yan J (2008). Principle component analysis and partial least squares. Appl. Multivar. Stat. Model..

[28] Nankaku M, Kanzaki H, Tsuboyama T, Nakamura T (2005). Evaluation of hip fracture risk in relation to fall direction. Osteoporos. Int..

[29] Palanca M, Perilli E, Martelli S (2020). Body anthropometry and bone strength conjointly determine the risk of hip fracture in a sideways fall. Ann. Biomed. Eng..

[30] Pomerantsev AL, Rodionova OY (2018). Multiclass partial least squares discriminant analysis: Taking the right way—A critical tutorial. J. Chemom..

[31] Poole K, Skingle L, Gee A, Turmezei T, Johannesdottir F, Blesic K, Rose C, Vindlacheruvu M, Donell S, Vaculik J, Dungl P, Horak M, Stepan J, Reeve J, Treece G (2017). Focal osteoporosis defects play a key role in hip fracture. Bone.

[32] Qasim M, Farinella G, Zhang J, Li X, Yang L, Eastell R, Viceconti M (2016). Patient-specific finite element estimated femur strength as a predictor of the risk of hip fracture: the effect of methodological determinants. Osteoporos. Int..

[33] Roche JJW, Wenn RT, Sahota O, Moran CG (2005). Effect of comorbidities and postoperative complications on mortality after hip fracture in elderly people: prospective observational cohort study. Br. Med. J..

[34] Rosipal, R., and N. Krämer. Overview and recent advances in partial least squares. In: Subspace, Latent Structure and Feature Selection (SLSFS 2005), edited by C. Saunders, M. Grobelnik, S. Gunn, and J. Shawe-Taylor. Lecture Notes in Computer Science, vol. 3940. Berlin: Springer, 2006. 10.1007/11752790_2.

[35] Sarkalkan N, Weinans H, Zadpoor AA (2014). Statistical shape and appearance models of bones. Bone.

[36] Sidey-Gibbons JAM, Sidey-Gibbons CJ (2019). Machine learning in medicine: a practical introduction. BMC Med. Res. Methodol..

[37] Slemenda, C., S. Cummings, E. Seeman, P. Lips, D. Black, and D. B. Karpf. Prevention of hip fractures: risk factor modification. *Am. J. Med.* 103(2A):65S–71S; discussion 71S–73S, 1997. 10.1016/s0002-9343(97)90028-0 9302898

[38] Sozen T, Ozisik L, Calik Basaran N (2017). An overview and management of osteoporosis. Eur. J. Rheumatol..

[39] Stegmann, M. B., R. Fisker, B. K. Ersbøll, H. H. Thodberg, and L. Hyldstrup. Active appearance models: theory and cases. In: Proceedings of the 9th Danish Conference on Pattern recognition and image analysis, pp. 49–57, 2000.

[40] Taddei F, Schileo E, Helgason B, Cristofolini L, Viceconti M (2007). The material mapping strategy influences the accuracy of CT-based finite element models of bones: an evaluation against experimental measurements. Med. Eng. Phys..

[41] Taylor M, Viceconti M, Bhattacharya P, Li X (2021). Finite element analysis informed variable selection for femoral fracture risk prediction. J. Mech. Behav. Biomed. Mater..

[42] Unnanuntana A, Gladnick BP, Donnelly E, Lane JM (2010). The assessment of fracture risk. J. Bone Jt. Surg. Am..

[43] Viceconti M, Qasim M, Bhattacharya P, Li X (2018). Are CT-Based finite element model predictions of femoral bone strengthening clinically useful?. Curr. Osteoporos. Rep..

[44] Viceconti M, Taddei F, Van Sint Jan S, Leardini A, Cristofolini L, Stea S, Baruffaldi F, Baleani M (2008). Multiscale modelling of the skeleton for the prediction of the risk of fracture. Clin. Biomech..

[45] Whitmarsh T, Fritscher KD, Humbert L, del Río Barquero LM, Roth T, Kammerlander C, Blauth M, Schubert R, Frangi AF (2012). Hip fracture discrimination from dual-energy X-ray absorptiometry by statistical model registration. Bone.

[46] Yang L, Udall WJM, McCloskey EV, Eastell R (2014). Distribution of bone density and cortical thickness in the proximal femur and their association with hip fracture in postmenopausal women: A quantitative computed tomography study. Osteoporos. Int..

