# Supplementary material for: Improving the Hip Fracture Risk Prediction with a Statistical Shape-and-Intensity Model of the Proximal Femur
Source: Ann Biomed Eng. 2022 Jan 19;50(2):211–21. doi: 10.1007/s10439-022-02918-z (PMC8803671; doi:10.1007/s10439-022-02918-z)
Supplement: Supplementary file 1 — Supplementary file1 (PDF 857 kb) [file 10439_2022_2918_MOESM1_ESM.pdf]

Supplementary Material

IMPROVING THE HIP FRACTURE RISK PREDICTION WITH A STATISTICAL SHAPE-AND-INTENSITY MODEL OF THE PROXIMAL FEMUR

Materials & Methods

Section 1 - The statistical shape analysis framework: *Deformetrica*

The performed Statistical Shape Analysis relied on the mathematical framework proposed by Durrleman et al. [3,5] and known as Deformetrica, which does not require landmarking using mathematical currents to represent and describe shapes. The main mathematical concepts of the approach will be presented in the following.

The current of a generic surface *S* (or curve *L*) is defined as the flux of a test vector field $\omega \in W$ across that surface (or curve). The resulting shape *T* of the surface *S* (or of curve *L*) is then uniquely characterised by the variations of the flux as the test vector field varies. W, in which ω varies, is a vector space generated by a Gaussian kernel K_W with width λ_W: $K_W(x, y) = e^{\frac{|x-y|^2}{\lambda_W^2}}$ for any points (x, y) and it is formally a reproducible kernel Hilbert space (r.k.h.s.).

The current of a surface (or curve) can be decomposed into an infinite set of Dirac delta currents, defined at each point of the surface (or curve) and oriented along the surface normal (or line tangent). Because computationally the surfaces (or curves) can be represented by discrete meshes (or polygonal lines), their current representation can be approximated by the finite sum:

$$S^i = \sum_k \delta_{x_k^i}^{\boldsymbol{a}_k^i}, \quad \text{(S1)}$$

with $\delta_{x_k^i}^{\boldsymbol{a}_k^i}$ being the so called Dirac delta current. A Dirac delta current can be seen as an infinitesimal vector that is concentrated at the barycentre of the mesh faces (or at the centre of each segment) x_k oriented along $\boldsymbol{a}_k^i$, the normal of the surface (or tangent to the line). The resolution of the currents representation is controlled by the above mentioned parameter λ_W, the width of the kernel K_W. The larger λ_W, the higher the spatial variation in the vector field and the coarser the resolution of the shape representation. Therefore, λ_W here defines the level of the shape details studied. Smaller λ_W values will allow to capture smaller differences between shapes, while larger values will discard them. Currents thus actually act as surrogate representations of shapes, characterizing them as distributions of shape features rather than as collections of points.

Once the input shapes have been modelled by means of currents, the shape modelling approach involves, according to the forward approach [4], the computation of the so called template $\bar{T}$, which represents the mean anatomical shape, and the simultaneous extraction of the transformation functions Φ_i, which map it towards

each ith patient-specific shape [3,8]. Each observation T_i, i.e. each subject-specific shape, is indeed described as a deformation of the template $\bar{T}$ plus some residuals ε_i, accounting for features not captured by the template nor by deformations:

$$T^i = \Phi^i \cdot \bar{T} + \varepsilon^i \qquad \text{(S2)}$$

The function Φ_i, defined using the Large Deformation Diffeomorphic Metric Mapping (LDDMM) approach[2,7], is parametrized by a time-varying velocity field uniquely characterized by an initial vector speed v_0^i:

$$v_0^i(x) = \sum_k K_V(x_k, x)\boldsymbol{\beta}_{x_k}^i. \qquad \text{(S3)}$$

v_0^i also belongs to a reproducible kernel Hilbert space V with kernel $K_V(x_k, x) = e^{\frac{|x_k - x|^2}{\lambda_V^2}}$ for any pair (x_k, x). λ_V is the kernel width, the x are the nodes of the surface mesh (or polygonal line), x_k the point position of the template Delta currents (also called control points). Hence, v_0^i is completely defined by the moment vectors $\boldsymbol{\beta}_{x_k}^i$ centred at the position x_k of the template delta currents, which drive the transformations of the template towards each shape and which contain the initial kinetic energy that is necessary to cover the path of a transformation from one Dirac delta current to the other. All the shape information present in the patients population, expressed as a unique deformation of the template shape, is thus contained in the patient-specific transformation functions $\Phi^i = f(\boldsymbol{\beta}^i)$ (Fig. S1). The template $\bar{T}$ and the deformations Φ^i towards each patient are estimated simultaneously by means of an alternate two-step minimization strategy [3]. λ_V, the width of the kernel K_V, defines the size of the area which is deformed consistently, i.e. the rigidity of the template deformation: the larger λ_V is, the stiffer the transformation will be, able to capture only the overall shape features variations.

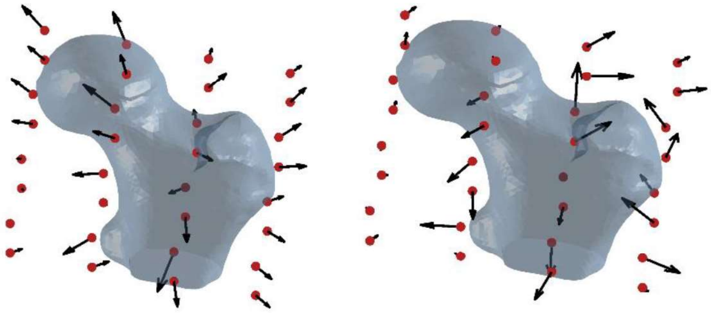

Fig. S1. Two patient-specific shapes, in blue, with superimposed, in black, the moment vectors, i.e. the vectors driving the template deformation towards each patient. They are centred on the same control points grid, defined with respect to the template.

Deformetrica, taking the shapes and the kernel widths λ_W and λ_V given as inputs equal to 10 and 28 mm respectively, computed and output the template shape, the reconstructed input shapes, the control point x_k coordinates and the corresponding moment vectors set mapping the template towards each patient-specific input shape.

Section 2 - Partial Least Square

PLS method identifies new bases of the space of predictors, which are relevant to an external response variable as well [9]. To do so, given two sets of centred variables, a generic predictors matrix $\boldsymbol{X}_C$ and a response

variables matrix $\boldsymbol{Y_C}$, the space of maximal covariance between them is computed. Mathematically, PLS estimates the weights vectors $\boldsymbol{r}$ and $\boldsymbol{s}$ which satisfy:

$$\max_{|\boldsymbol{r}||\boldsymbol{s}|=1} cov(\boldsymbol{X_C r}, \boldsymbol{Y_C s}) = \max_{|\boldsymbol{r}||\boldsymbol{s}|=1} var(\boldsymbol{X_C r}) corr(\boldsymbol{X_C r}, \boldsymbol{Y_C s})^2 var(\boldsymbol{Y_C s}), \quad \text{(S4)}$$

as opposed to PCA, which solves $max_{|\boldsymbol{r}|=1} var(\boldsymbol{X_C r})$. The PLS modes $\boldsymbol{t}$ and $\boldsymbol{u}$ are then found weighting $\boldsymbol{X_C}$ and $\boldsymbol{Y_C}$ by $\boldsymbol{r}$ and $\boldsymbol{s}$ within an iterative process (Table S1) which loops until all the PLS modes are extracted and where, at each step, the variance explained by $\boldsymbol{r}$ and $\boldsymbol{s}$ is removed from the original data (deflation). Mathematically, this can be written as:

$$\boldsymbol{X_C} = \boldsymbol{T}\boldsymbol{P}^T + \boldsymbol{E} \qquad \text{(S5)}$$

$$\boldsymbol{Y_C} = \boldsymbol{U}\boldsymbol{Q}^T + \boldsymbol{F} \qquad \text{(S6)}$$

$\boldsymbol{T}$ and $\boldsymbol{U}$ are the PLS components matrices, $\boldsymbol{P}$ and $\boldsymbol{Q}$ contain the PLS modes, or loadings (contrary to PCA loadings, the PLS ones are not necessary orthogonal); $\boldsymbol{E}$ and $\boldsymbol{F}$ are residual matrices. The PLS components also need to satisfy the regression equation $\boldsymbol{U} = \boldsymbol{TD} + \boldsymbol{G}$, $\boldsymbol{D}$ being a diagonal matrix of weights and $\boldsymbol{G}$ the matrix of residuals.

Table S1. PLS space decomposition (PLS1 algorithm).

Inputs: $\boldsymbol{X_C}$, $\boldsymbol{Y_C}$, number of PLS modes p to extract ($p = N - 1$)

for $n = 1$ to p ***do***

$\boldsymbol{r}^n \leftarrow first\ eigenvector\ of\ \boldsymbol{X}_C^{n\,T}\boldsymbol{Y}_C^n\boldsymbol{Y}_C^{n\,T}\boldsymbol{X}_C^n\ (from$ [6]$)$

$\boldsymbol{t}^n \leftarrow \boldsymbol{X}_C^n\boldsymbol{r}^n/|\boldsymbol{r}^n| \quad n^{th} PLS\ component\ of\ \boldsymbol{X_C}$

$\boldsymbol{s}^n \leftarrow \boldsymbol{Y}_C^n\boldsymbol{t}^n/(\boldsymbol{t}^{n\,T}\boldsymbol{t}^n)$

$\boldsymbol{u}^n \leftarrow \boldsymbol{Y}_C^n\boldsymbol{s}^n/|\boldsymbol{s}^n| \quad n^{th} PLS\ component\ of\ \boldsymbol{Y_C}$

$\boldsymbol{p}^n \leftarrow \boldsymbol{X}_C^n t^n/(\boldsymbol{t}^{n\,T}\boldsymbol{t}^n) \quad n^{th} PLS\ loading\ of\ \boldsymbol{X_C}$

$\boldsymbol{q}^n \leftarrow \boldsymbol{Y}_C^n\boldsymbol{u}^n/(\boldsymbol{u}^{n\,T}\boldsymbol{u}^n) \quad n^{th} PLS\ loading\ of\ \boldsymbol{Y_C}$

$\boldsymbol{X}_C^{n+1} \leftarrow \boldsymbol{X}_C^n - \boldsymbol{t}^n\boldsymbol{p}^{n\,T} \quad deflation\ of\ \boldsymbol{X_C}$

$\boldsymbol{Y}_C^{n+1} \leftarrow \boldsymbol{Y}_C^n - \boldsymbol{t}^n[\boldsymbol{t}^{n\,T}\boldsymbol{Y}_C^n/(\boldsymbol{t}^{n\,T}\boldsymbol{t}^n)] \quad deflation\ of\ \boldsymbol{Y_C}$

end

Section 3 – Statistical models construction

PLS performs dimensionality reduction, and here was applied to identify the main shape and BMD distribution attributes simultaneously relevant to the surrogate fracture risk. Given an input data matrix *X* (e.g. the shape or intensity matrix) and a response variable *Y* (e.g. the fracture status array), PLS computes the directions in the space of the variables, termed PLS modes, where the covariance between *X* and *Y* is maximal. By projection of the original data matrix *X* onto the PLS modes, the PLS components are identified which best account for the variance of *X*, *Y* and the correlation between the two. Taking the surrogate fracture risk as the response variable, PLS allowed to identify the main shape and BMD distribution attributes simultaneously relevant to the surrogate fracture risk too.

The PLS-based Statistical Shape Model (SSM) and the Statistical Intensity Model (SIM) can be represented with the following expressions:

$$\beta^i = \bar{\beta} + \sum_{j=1}^{m} t_j^{S,i}\, p_j^S \qquad \text{(S7)}$$

$$g^i = \bar{g} + \sum_{j=1}^{m} t_j^{G,i}\, p_j^G. \qquad \text{(S8)}$$

Where β^i and g^i refer to the ith patient shape and intensity, $\bar{\beta}$ and $\bar{g}$ to the average shape and intensity, p_j^S and p_j^G are the PLS modes, $t_j^{S,i}$ and $t_j^{G,i}$ the PLS components. In order to build the Statistical Shape-Intensity Model (SSIM), in accordance with Cootes and co-workers [1], a concatenated shape and intensity PLS components matrix $\boldsymbol{T}$ was built up, based upon the only shape and only intensity PLS components previously computed as follows:

$$\boldsymbol{T} = \begin{pmatrix} \boldsymbol{W}_{PLS}\, \mathbf{T}^{S'} \\ \mathbf{T}^{G'} \end{pmatrix}, \qquad \text{(S9)}$$

where $\boldsymbol{W}_{PCA}$ is a weighting factor matrix, expressed as:

$$\boldsymbol{W}_{PCA} = r\, \boldsymbol{I} \qquad \text{(S10)}$$

where r is the ratio between the total variance in only intensity and the total variance in only shape PCA modes, and $\boldsymbol{I}$ is the identity matrix. The matrices $\mathbf{T}^S$ and $\mathbf{T}^G$ gather the shape and intensity components (gathering the t_j^S and t_j^G arrays). By applying a third PLS onto the concatenated matrix $\boldsymbol{T}$, combined shape-intensity PLS modes were identified. Subsequently, by projection of the matrix $\boldsymbol{T}$ onto the identified PLS subspace, the patient-specific combined shape-intensity PLS components were obtained.

Results

Statistical models explained variance

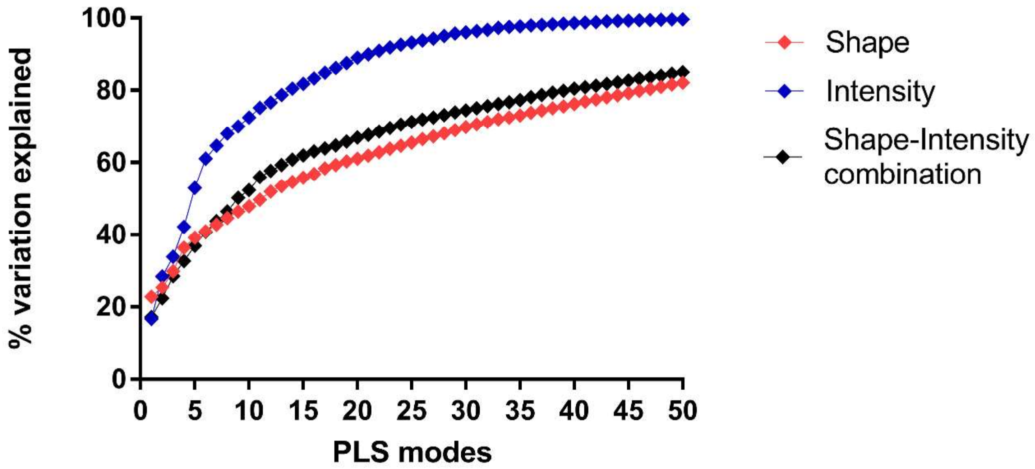

Figure S2. Comparison between the % variation of the shape, intensity and their combination for the SSM, SIM and SSIM.

Logistic regression models

Considering two predictors x_1 and x_2 and one binary response variable Y, which can be denoted as $p = P(Y = 1)$, a logistic regression model establish a linear relationship between the predictors and the log-odds l of the event $Y = 1$:

$$l = \beta_0 + \beta_1 x_1 + \beta_2 x_2$$

With l being the logarithm of the odds $\frac{p}{1-p}$: $l = log\frac{p}{1-p}$, β being the regression model coefficients.

In the following, the different regression models coefficients are provided for the statistical shape, intensity and shape-intensity models.

Table S1. SSM-based logistic models. Logistic regression models coefficients for the full model, i.e. built on the PLS carried out on the whole cohort, and the logistic models coefficients as coming from the 10 folds. In the 'Average' column, the average coefficients across the 10 folds ar reported.

	Full model	Cross-validation 10 folds										Average
β_0	-0.003	0.008	-0.035	-0.021	0.073	0.173	0.021	0.047	-0.026	-0.046	-0.031	0.016
β_1	0.356	0.400	0.454	0.403	0.392	0.559	0.424	0.335	0.392	0.364	0.474	0.420
β_2	0.329	0.321	0.349	0.294	0.373	0.464	0.320	0.383	0.397	0.398	0.361	0.366

Table S2. SIM-based logistic models. Logistic regression models coefficients for the full model, i.e. built on the PLS carried out on the whole cohort, and the logistic models coefficients as coming from the 10 folds. In the 'Average' column, the average coefficients across the 10 folds are reported.

	Full model	Cross-validation 10 folds										Average
β_0	-0.014	-0.429	-0.169	0.006	0.388	0.233	0.316	-1.353	-0.099	-0.018	-0.050	-0.118
β_1	0.297	0.205	0.247	0.271	0.236	0.287	0.251	0.386	0.282	0.197	0.301	0.266
β_2	0.960	0.614	0.786	0.998	0.774	0.723	0.806	1.148	0.846	0.615	0.751	0.806

Table S3. SSIM-based logistic models. Logistic regression models coefficients for the full model, i.e. built on the PLS carried out on the whole cohort, and the logistic models coefficients as coming from the 10 folds. In the 'Average' column, the average coefficients across the 10 folds are reported.

	Full model	Cross-validation 10 folds										Average
β_0	0.352	-2.285	-0.803	-6.987	-3.862	-2.881	2.772	6.213	-2.898	-1.185	-2.319	-1.424
β_1	5.694	5.221	4.729	5.392	4.763	5.644	4.697	5.164	4.921	5.193	5.237	5.096
β_2	7.036	5.767	5.642	6.470	5.116	6.322	6.098	6.422	5.269	6.213	5.062	5.838

Table S4. Clinical information for the subjects included in the cohort. Fracture status is reported as F for fractured patients and C for control ones. aBMD is referred to the neck region.

Patient ID	Age	Height (cm)	Weight (kg)	Fracture Status	aBMD (g/cm^2)	T-score
1	62.1	166.7	56.5	F	0.59	-2.7
2	63.5	166.1	53.5	C	0.81	-1.5
3	59.8	168.1	91.7	F	0.76	-1.9
4	81.4	146.7	31	F	0.46	-4.2
5	71.1	168.5	81	F	0.74	-0.3
6	60	166.3	92.7	C	0.97	-0.3
7	87.6	149.8	64	F	0.43	-3.2
8	81	153.2	65.1	F	0.73	-2.9
9	81.7	148.9	56.1	F	0.64	-2.9
10	80.4	150.8	57.4	C	0.66	-1.4
11	88.2	157	54.2	F	0.81	-2.6
12	71.7	159.4	62.5	F	0.63	-0.9
13	80.2	161	70.5	F	0.91	-1.3
14	57.8	152.3	34.2	F	0.84	-2.6
15	70.8	166.5	82.4	C	0.70	0.9
16	87.7	166.5	82.4	C	0.95	-2.2
17	71.1	156.2	63.9	C	0.62	-2.8
18	63.7	168.4	51.5	F	0.83	-3.3
19	81.1	163.1	72.5	C	0.62	-1.5
20	62.1	166.6	101.1	F	0.79	-1
21	57.7	168.4	80.7	F	0.86	-0.8
22	83	157.3	50	F	0.94	-3.1
23	70.5	150.7	43.4	F	0.57	-3.8
24	84.7	154	49	C	0.62	-2.5
25	67.5	155.7	84.1	F	0.54	0.1
26	55.9	169.3	76.3	C	0.86	-2.3
27	60.1	150.1	44.9	C	0.67	-1.6
28	67.4	155.8	77.9	C	0.87	-0.2
29	85.1	151.5	65.7	C	0.84	-1.9
30	64	151.3	42.8	C	0.84	-2.5
31	59.3	160.5	53.9	F	0.76	-1.2
32	71.2	169.6	71.8	F	0.80	-3
33	84.9	153	58.5	C	0.66	-1.9
34	78.5	155.7	60.9	F	0.68	-2.9
35	62.8	159.5	86.4	C	0.54	1
36	86.6	160.5	40.1	F	1.30	-3.1
37	61.5	160.7	58.1	C	0.45	-1.1
38	80.6	160.6	66.6	C	0.82	-0.6
39	63.9	162.6	58.5	F	0.83	-1.9
40	87.5	158.6	50.3	F	0.80	-3.1
41	86.2	150.6	58.8	F	0.84	-2.2
42	66.3	155.1	67.8	C	0.76	-0.2
43	78.9	155.1	46.5	F	0.95	-3.4
44	82	157.5	60.6	F	0.52	-2.9
45	74.5	157.1	79.5	F	0.60	-0.4
46	73.2	153.3	63.9	F	0.75	-1.2
47	78.1	159.1	59.4	C	1.16	-1.5

48	84.7	154.3	55	C	0.83	0.2
49	68.1	152.5	60.8	C	0.83	-1.5
50	80.7	156.5	61.6	C	0.94	-0.6
51	70.8	156	62.3	F	0.86	-2
52	78.9	156.6	48.1	C	0.66	-2.2
53	74.1	158	76.7	C	0.69	-0.1
54	79	156.9	62	F	0.70	-2.3
55	73.6	157	64.3	F	0.73	-2.5
56	75.4	152.5	62	C	0.67	-1.9
57	58.4	157.7	44.9	F	0.70	-3
58	70.9	155.4	65.8	C	0.92	-1.1
59	86	148.7	66.9	F	0.95	-3
60	77.7	173.1	65.2	F	0.54	-1.2
61	86.6	150.9	61.1	C	0.43	-1.9
62	75.2	159.5	65.3	F	0.55	-1.4
63	73	156.6	63.9	C	0.70	-0.9
64	78.2	150.1	69	C	0.90	-2.4
65	80.7	153	72.8	C	0.66	-1.2
66	81.7	168.3	68.2	C	0.77	-0.7
67	74.8	154	64.8	C	0.73	-1
68	81.9	170.2	77.3	F	0.76	-0.9
69	82.7	160.5	60.3	F	0.78	-1
70	88.7	145	48.1	F	0.76	-5
71	67.4	152	48.2	C	0.46	-2.3
72	80	157.2	61.1	F	0.66	-0.9
73	78.4	162.6	65	F	0.77	-2.9
74	64.1	162.5	52.4	C	0.56	0
75	68.1	165	58.8	C	0.94	0
76	65.1	148.8	76.8	C	0.91	-0.3
77	80.8	153.6	50.7	F	0.86	-2.6
78	77.7	168.1	73.6	C	0.55	-0.5
79	84	162	59	C	0.83	-2
80	60.2	157	50.2	C	0.59	-0.6
81	91	151	45	C	0.90	-2.9
82	78.8	164.3	53.9	F	0.60	-2.9
83	80.9	157.1	54.2	C	0.70	-0.2
84	76.4	162.6	79.8	F	0.66	-1.3
85	76.9	162	63.6	C	0.95	0.9
86	78.7	157.9	83.4	F	0.72	-0.5
87	70	160	75.4	F	1.13	-0.1
88	87.5	160	73	C	0.78	-0.2
89	73.3	163	81.6	C	0.85	-0.4
90	80	151.4	68.3	F	0.90	-0.2
91	76.7	160	86.8	C	0.73	1.1
92	74.6	159.5	48.9	C	0.87	-1.6
93	83.1	154	63.8	C	0.71	-0.6

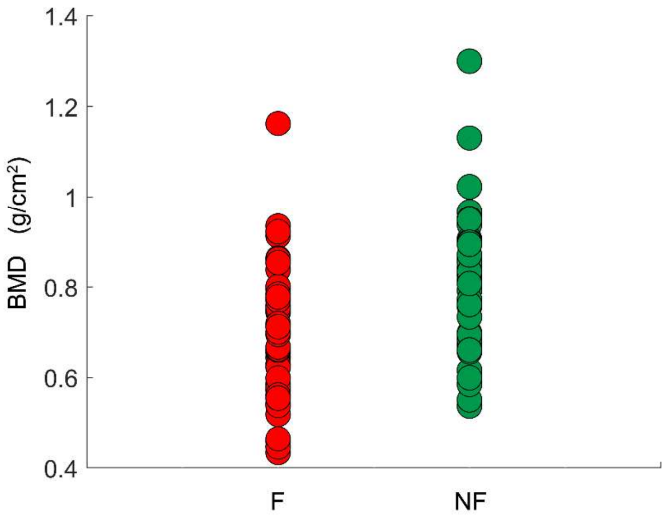

Figure S3. aBMD values at the femoral neck for the whole cohort depicted in red or green according to the fracture status of the patients (F: Fractured, red; NF: Non fractured, green).

References

1. Cootes, T. F., G. J. Edwards, and C. J. Taylor. Active appearance models. In: Lecture Notes in Computer Science (including subseries Lecture Notes in Artificial Intelligence and Lecture Notes in Bioinformatics). 1998, pp. 484–498.
2. Durrleman, S. Statistical models of currents for measuring the variability of anatomical curves , surfaces and their evolution. , 2011.
3. Durrleman, S., X. Pennec, A. Trouvé, and N. Ayache. Statistical models of sets of curves and surfaces based on currents. *Med. Image Anal.* 13:793–808, 2009.
4. Durrleman, S., X. Pennec, A. Trouvé, P. Thompson, and N. Ayache. Inferring brain variability from diffeomorphic deformations of currents: An integrative approach. *Med. Image Anal.* 12:626–637, 2008.
5. Durrleman, S., M. Prastawa, N. Charon, J. R. Korenberg, S. Joshi, G. Gerig, and A. Trouvé. Morphometry of anatomical shape complexes with dense deformations and sparse parameters. *Neuroimage* 101:35–49, 2014.
6. Höskuldsson, A. PLS regression methods. *J. Chemom.* , 1988.doi:10.1002/cem.1180020306
7. Mansi, T. Image-Based Physiological and Statistical Models of the Heart – Application to Tetralogy of Fallot. , 2010.
8. Mansi, T., I. Voigt, B. Leonardi, X. Pennec, S. Durrleman, M. Sermesant, H. Delingette, A. M. Taylor, Y. Boudjemline, G. Pongiglione, and N. Ayache. A statistical model for quantification and prediction of cardiac remodelling: Application to tetralogy of fallot. *IEEE Trans. Med. Imaging* , 2011.doi:10.1109/TMI.2011.2135375
9. Rosipal, R., and N. Krämer. Overview and recent advances in partial least squares. , 2006.doi:10.1007/11752790_2
